# Hair Follicle Melanocyte Cells as a Renewable Source of Melanocytes for Culture and Transplantation

**Published:** 2008-01-09

**Authors:** Ho Kwon, Perry H. Liu, Dae-Hyun Lew, Emi Nishimura, Dennis P. Orgill

**Affiliations:** Tissue Engineering and Wound Healing Laboratory, Division of Plastic & Reconstructive Surgery, Brigham and Women's Hospital, Harvard Medical School, Boston, MA

## Abstract

**Objective:** Advances in melanocyte culture techniques have not yet led to reliable clinical methods for treating hypopigmentation disorders. We hypothesized that melanocytes harvested from plucked hair follicles may provide a renewable source of melanocytes for the treatment of hypopigmentation. **Methods:** Hairs with attached cells from the follicles were plucked from Yucatan pigs and implanted in a collagen-glycosaminoglycan matrix for either immediate or delayed implantation into full-thickness excisional porcine wounds. Wounds were allowed to heal and were biopsied at 2 and 4 weeks, respectively. **Results:** Fully healed wounds with transplanted hair follicles showed central areas of dark pigmentation corresponding to the location of implanted hair follicles. Corresponding collagen-glycosaminoglycan matrix wounds showed no central areas of pigmentation. **Conclusions:** Hair follicle--derived melanocytes may potentially serve as a renewable source of pigment-producing cells for treating hypopigmentation disorders.

Skin color is due to a complex interaction between ambient light and cutaneous biomolecules. Arterialized blood flow brings hemoglobin near the skin surface and gives a red hue to the skin. Melanocytes produce a dark polymer known as melanin, which gives skin its dark color. Derived from neural crest cells, melanocytes have physical connections to keratinocytes through which melanin granules are deposited. Skin pigmentation varies greatly depending on the location on the body or the race of an individual.

Pigmentation disorders are common and can result in either an over- or underexpression of melanin. Hyperpigmentation disorders are frequently seen after trauma and burns and can result in a skin color dramatically darker than the surrounding uninjured skin. Loss of melanocytes or decreases in melanin production leads to pigmentation disorders. These are most commonly seen in aging hair because it becomes gray or white over time. In addition, burns, laser therapy, and autoimmune disorders (eg, vitiligo) can also result in skin hypopigmentation.

Until the 1980s, it was not possible to grow melanocytes in vitro. This view changed when Eisinger and Marko$^{1}$ demonstrated that melanocytes could be cultured and serially passaged using culture medium containing 12-*O*-tetradecanoyl-phorbol-13-acetate (TPA). However, phorbol esters, including TPA, were also demonstrated to be toxic to keratinocytes and limited their usefulness for in vitro melanocyte studies and clinical applications. Although there have been no reports of melanocyte mutations, malignant transformations of melanocytes grown using phorbol esters continue to be of concern.

Lerner et al^2^ demonstrated that the use of cultured and noncultured epithelial melanocytes could be effective in the treatment of patients affected by piebaldism. Falabella et al^3^ further demonstrated that culture medium could be developed to culture epidermal melanocytes in vitro without the use of potentially oncogenic reagents such as phorbol esters. These methods allow epidermal melanocytes to be incorporated into artificial skin constructs, to repopulate skin melanocytes using a cell spray apparatus, or permit their autotransplantation from shaved skin biopsies.^4^–^8^ These experiments resulted in the development of currently practiced techniques for treating pigmentation disorders such as vitiligo and leukoderma.^9^ These treatment modalities include epithelial sheet grafting, mini grafting, epidermal grafting with suction blisters, and transplantation of in vitro cultured skin melanocytes via skin blister injections. Despite these ongoing efforts, no widely accepted method is currently available for treating clinical hypopigmentation.

Current methods use epidermal melanocytes requiring at least a small skin biopsy. This limits immediate autologous melanocyte transplantation and necessitates in vitro melanocyte culture before transplantation. Rather than depending on the limited supply of epidermal melanocytes from skin biopsies, we propose to use melanocytes found in the upper outer hair follicle root sheath. It has been known that these melanocytes are capable of migrating from these reservoirs to pigment their respective hair follicles. Nishimura et al^10^ further demonstrated in a mouse model that melanocyte stem cells can be found in the lower permanent portion of the hair follicle. These stem cells were shown to be able to migrate to “niches” lacking melanocytes and repopulate them. They further suggest that these stem cells could potentially be the source for both hair follicle and epidermal melanocytes. In this study, we use plucked hair follicle melanocytes to show repigmentation in a porcine model.

## MATERIALS AND METHODS

### Porcine model

All animals were treated in an AAALAC-approved facility under an approved animal protocol. Three 2-month-old Yucatan mini pigs (Charles River, Wilmington, MA) were housed and treated in the animal care facility at Brigham and Women's Hospital (Boston, MA) for at least 3 days before the start of each experiment. After acclimation, the pigs were sedated (telazol 5 mg/kg IM and xylazine 2 mg/kg IM) and transported into our animal operating room. General anesthesia was administered via snout mask using halothane (1.5%–3.0%), oxygen (4–6 L/min), and nitrous oxide (2–3 L/min) and monitored using continuous pulse oximetry. The dorsum and scalp of the pig was scrubbed and painted 3 times using a betadine solution. Hair follicles were then plucked from these areas and immediately transferred into an enriched Gibco keratinocyte serum-free media (S-FM; Invitrogen, Carlsbad, CA).

Sixteen 3.0×3.0-cm full-thickness skin wounds were incised on the paraspinous region of the dorsum. Hemostasis was obtained with direct pressure and electrocautery. Collagen-glycosaminoglycan (GAG) matrices, either with or without hair follicles, were sutured in place and stabilized using a semiocclusive polyurethane dressing (Tegaderm, St. Paul, MN), gauze, and a protective cotton vest.

### Harvesting hair follicles

**Figure 1:**
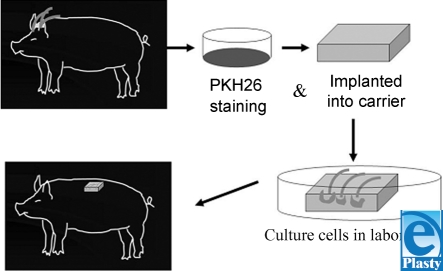
Diagram depicting the harvesting and transplantation of plucked hair follicles.

Anagen phase hair follicles were plucked from the scalp and back of a Yucatan pig. All plucked hair follicles were immediately transferred into an enriched Gibco keratinocyte S-FM and subsequently examined under a microscope to ensure that hair root bulbs and sheath were intact. We also removed excess hair root debris from around each bulb. Hair follicles were then implanted into collagen-GAG matrices after being stained with a PKH26 fluorescent dye for cell tracking, and either immediately placed into newly ceated full-thickness skin wounds or grown in cell culture media for 2 weeks before implantation. After the matrices containing 3 hair follicles were applied to the dorsal wounds of each pig, they were covered with Tegaderm and followed daily until complete wound healing (Fig 1).

### Collagen-CAG matrices

Porous matrices were prepared using a procedure initially described by Yannas et al.^11^ A collagen dispersion was prepared using 0.5% bovine hide collagen (Sigma Chemical, St. Louis, MO) in 0.05 M acetic acid (pH 3.2) with chondroitin 6-sulfate (sodium salt, Type C, Sigma Chemical, St. Louis, MO) in a custom-built blender with an internal cooling apparatus. This was poured into a 3-mm thick stainless steel pan and transferred to a freeze dryer with a shelf temperature of −r°40°C (Virtis Unitop 800, Gardiner, NY) for 20 hours. After lyophilization, matrices were cross-linked using a dehydrothermal treatment at 105°C for 24 hours, followed by UV irradiation at 254 nm at 4510 μW/cm^2^ for 45 minutes. Matrices were then cut into 3.0×3.0-cm squares under sterile conditions.

### Cell culture

We used an enriched Gibco keratinocyte S-FM for storing harvested hair follicles, hydrating collagen-GAG matrices before follicle placement and coverage of wounds, and culturing follicular melanocytes in vitro. This media consists of using keratinocyte S-FM supplemented with pituitary extract (Invitrogen, Carlsbad, CA), epidermal growth factor (EGF) (R&D Systems, Minneapolis, MD), pen-strep-Glutamine (PSQ) (Shimazu, Kyoto, Japan), hFGF, and TPA (Sigma, St. Louis, MO).

### PKH26 fluorescent red stain

Harvested hair follicles were rinsed using a phosphate buffered saline solution and then stained using PKH26 fluorescent cell linker (Sigma, St. Louis, MO) for cell tracking. One group (*n* = 8) was immediately placed on to collagen-GAG matrices for immediate transplantation, whereas a second group (*n* = 8) was grown in enriched keratinocyte S-FM for 2 weeks before transplantation.

### Melanocyte transplantation

Follicular melanocytes using 3.0×3.0-cm collagen-GAG matrices as a delivery agent were transplanted into newly created 3-cm^2^ full-thickness skin wounds on day 0 and day 14. Follicular melanocytes that were transplanted on day 0 were stained after being harvested and immediately reimplanted into dorsal wounds (*n* = 8). Melanocytes transplanted on day 14 were grown in vitro for 2 weeks before transplantation (*n* = 8). All wounds were covered with a semiocclusive polyurethane dressing and followed daily until complete wound healing.

### Wound harvesting and histology

Wounds were harvested on day 14 and day 28 under general anesthesia with a 1-cm margin of normal tissue. After biopsies were taken on day 14, the resulting wounds were covered with isotonic sodium chloride solution–soaked collagen-GAG matrices and using the same polyurethane dressing. After biopsies were taken on day 28, each animal was euthanized. One set of biopsy specimens was stored in 10% formalin, transferred to cassettes, embedded, sectioned, and stained with hematoxylin and eosin. Two observers, both blinded to the specimen groups, independently counted the number of melanocytes present in each wound. The second set of biopsy specimens was frozen immediately using liquid nitrogen and stained with a DAPI solution in phosphate buffered saline for visualization under a fluorescent microscope (Axioplan 2 Imaging, Zeiss, TMS, Nikon). Images of histological slides were taken using an inverted microscope at 200× magnification. Cell counting was performed using the same microscope under 400× magnification.

### Results

Hair follicle melanocytes begin to proliferate in the early Anagen II stage and continue through the Anagen III stage of the hair growth cycle. After harvesting, melanocytes were either grown in vitro using enriched keratinocyte S-FM culture for 2 weeks or transplanted immediately.

**Figure 2:**
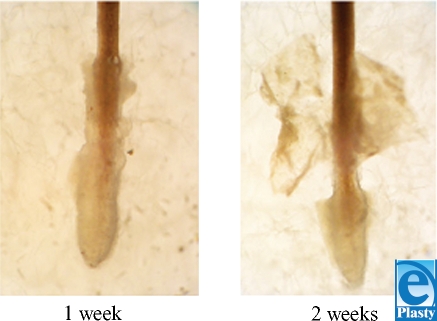
Hair follicles grown in vitro. Hair follicle stalks demonstrate darker pigmentation at after 2 weeks of incubation in enriched keratinocyte serum-free media.

Among the cultured melanocytes, a pigmented area was identified around the implanted hair follicle in the matrix before transplantation (Fig 1). This area corresponds to an area of potential melanocyte migration from the hair follicle bulb. We could see the pigmented area around the base of cultured hair follicles on both day 7 and day 14. After the transplantation, the dark-brown-colored area (arrow) is still visible around the transplanted hair follicle (Fig 2).

**Figure 3:**
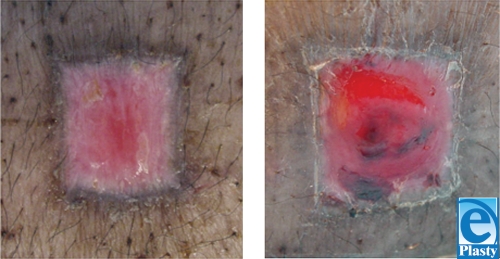
Healing wounds 2 weeks postoperatively. Melanocyte-transplanted wounds (transplantation site) (right) demonstrate central areas of pigmentation, whereas nontransplanted wounds contain no pigmentation (control site) (left).

When hair follicles were transplanted immediately after harvest and allowed to heal, we were also able to identify the pigmented area 2 weeks posttransplantation. This pigmented area is similar to that seen among cultured melanocytes. We could see a dark-brown area around the transplanted hair follicles, whereas no pigmentation was observed in the nontransplanted wound. Over time, as the surgical wounds healed, this dark-brown color became more pronounced (Fig 3).

**Figure 4:**
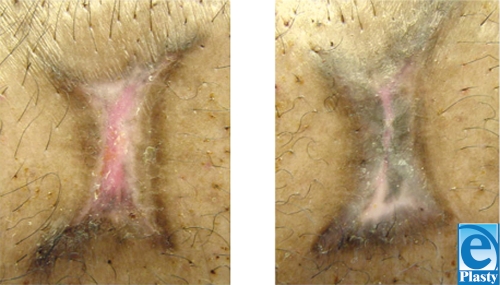
Healed wounds 4 weeks postoperatively. Transplanted wounds (transplantation site) (right) exhibit darker central areas of pigmentation than nontransplanted wounds (control site) (left).

After complete healing (4 weeks posttransplantation), central areas of pigmentation observed in hair follicle transplanted wounds appeared even darker and thicker than their counterparts at 2 weeks posttransplantation (Fig 4). Despite marked wound contraction, a rim of nonpigmented tissue visibly demarcated the central area of hair follicle melanocyte pigmentation from the rim of epidermal melanocyte pigmentation surrounding each wound.

**Figure 5:**
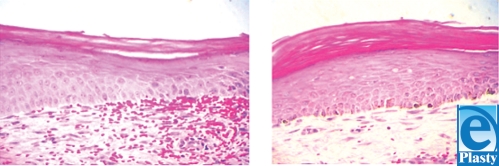
Histological sections of control wound (nontransplantation wound: 0.2 ± 0.4 pigmented melanocytes) (left) and melanocyte-transplanted wound (transplant wound: 6.0 ± 1.4 pigmented melanocytes) (right) harvested at 4 weeks postoperatively.

Histological sections of the wound show that a large number of melanocytes per high-power field can be seen in transplanted wounds (Fig 5). The study wound contained 6.0 ± 1.4 pigmented melanocytes per high-power field, whereas control wounds had only 0.2 ± 0.4 pigmented melanocytes.

**Figure 6:**
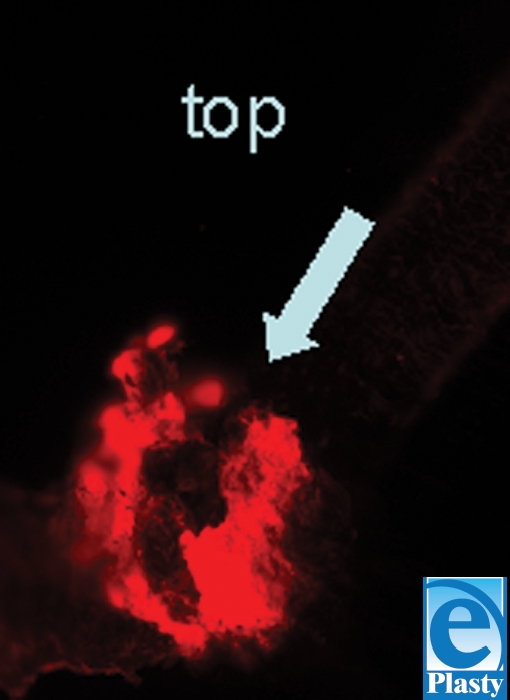
PKH26-stained follicular melanocytes in the hair bulb as seen under a fluorescent microscope using a Texas red view.

**Figure 7:**
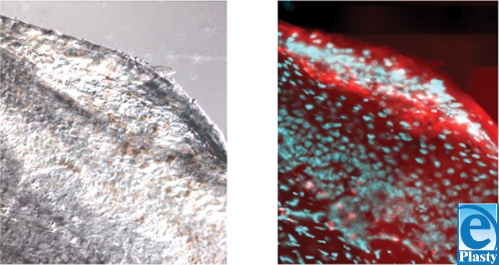
DIC views revealing dark-colored melanocytes (left) and overlapping Texas red and DAPI views (right) of histological specimens from transplanted wounds.

To distinguish follicular melanocytes from their epidermal counterparts, plucked hairs were stained with PKH26 fluorescent dye for cell tracking before transplantation (Fig 6). We detected PKH26-positive cells containing melanin pigments in the transplanted areas, indicating that melanocytes in the pigmented area are derived from the follicle implant (Fig 7).

## DISCUSSION

We were able to demonstrate that melanocytes from plucked hair follicles were viable and proliferated both in vitro and in vivo. Unlike epidermal skin melanocytes, plucked hair follicular melanocytes are much easier to harvest, are less invasive, and lack donor site morbidity in terms of scarring and color changes.

In the treatment of severe burns, cultured epidermal autografts have frequently shown irregular areas of hypopigmentation. Melanocytes in cultured epithelial grafts are usually depleted with serial subcultivation and cryopreservation.^12^ Transferred using either an artificial collagen matrix--containing hair follicle melanocytes or the plucked hair bulbs themselves as a micrograft into normal skin would provide a simple, effective, and low-morbidity alternative to current practices. This would eliminate the need for invasive tissue sampling, laborious melanocyte extraction procedures, and the use of potentially toxic phorbol-based melanocyte culture media. Hair follicle--derived melanocyte stem cells are flexible, adaptable, and ready-to-use immediately.

We showed that follicular melanocytes, in addition to their ability to migrate up a hair follicle to give it a characteristic color, are capable of migrating out into the surrounding melanocyte-deficient skin.^13^,^14^ Transplanted follicular melanocytes, labeled using PKH26 fluorescent cell linker dye, can be found in the healed wound. Healed wounds, which were treated with collagen-GAG matrices alone, did not show central areas of pigmentation and no melanocytes were observed on histology. This indicates that the observed melanocytes are of follicular rather than epidermal origin. This result is consistent with the findings of Medalie et al,^15^ where transplanted epidermal melanocytes repopulated portions of acellular dermal substitutes but stopped at the edges of the graft.

Since melanoblasts on the outer root sheath of the follicle include a stem cell population, we have shown that it is feasible to harness this renewable source of melanocytes for clinical and research purposes. More adaptable than differentiated melanocytes, these stem cells are a dependable source of melanocytes for transplantation, studying their biology, and advancing our ability to manipulate their melanin expression.

The renewable and autologous nature of this technique makes melanocyte stem cells more conducive to both culturing and pigment manipulation. It could translate clinically into the development of better treatments for all iatrogenic, acquired, or hereditary hair and skin pigmentation disorders. Failed melanocyte transplants resulting in pigmentation irregularities could be salvaged through follicular melanocyte transplants. Having been shown to affect keratinocyte differentiation and proliferation, melanocytes could potentially improve both wound healing and pigmentation.^16^ Boyce et al^17^ further showed that cultured skin substitutes, which contained melanocytes, improved both wound contracture and pigmentation. In melanocyte-deficient skin, such as that of a healed burn wound or hypopigmented laser resurfaced skin, melanoblasts in hair follicles could potentially reconstitute the entire melanocyte population.

Although we cannot conclusively prove that the melanocytes grow out from the hair follicle, the wound photographs and staining results are consistent with this hypothesis. In addition, significant contraction observed in these cutaneous wounds is also a limitation of this study. Navsaria et al^18^ have used micrografts to restore epithelium to collagen-GAG matrices in humans with success, but no mention was made about the issue of pigmentation. More detailed studies will be required to determine the optimal transplantation characteristics of melanocytes attached to hair follicles.
